# Activity Variation of *Phanerochaete chrysosporium* under Nanosilver Exposure by Controlling of Different Sulfide Sources

**DOI:** 10.1038/srep20813

**Published:** 2016-02-11

**Authors:** Zhi Guo, Guiqiu Chen, Lingzhi Liu, Guangming Zeng, Zhenzhen Huang, Anwei Chen, Liang Hu

**Affiliations:** 1 College of Environmental Science and Engineering, Hunan University, Changsha 410082, P.R. China; 2 Key Laboratory of Environmental Biology and Pollution Control (Hunan University), Ministry of Education, Changsha 410082, P.R. China; 3 College of Resources and Environment, Hunan Agricultural University, Changsha 410128, P.R. China

## Abstract

Due to the particular activation and inhibition behavior of silver nanoparticles (AgNPs) on microbes at various concentrations, it’s crucial to exploit the special concentration effect in environment. Here, we studied the viability variation of *Phanerochaete chrysosporium* (*P. chrysosporium*) under exposure to citrate-coated AgNPs (Citrate-AgNPs) in the presence of different sulfide sources (an inorganic sulfide, NaHS and an organic sulfide, thioacetamide (TAA)). The results indicated that both NaHS and TAA can promote activation of *P. chrysosporium* by Citrate-AgNPs at a higher concentration, which was initial at toxic level. Treatment with various concentrations of Citrate-AgNPs (0–9 mg/L) demonstrated a maximum activation concentration (MAC) at 3 mg/L. With the increase in sulfide concentration, MAC transferred to higher concentration significantly, indicating the obvious “toxicity to activation” transformation at a higher concentration. Ag^+^ testing exhibited that variations in sulfide-induced Ag^+^ concentration (3−7 μg/L Ag^+^) accounted for the “toxicity to activation” transformation. In addition, the similar results were observed on antibacterial application using *Escherichia coli* as the model species. Based on the research results, the application of this transformation in improving antibacterial activity was proposed. Therefore, the antibacterial activity of AgNPs can be controlled, even at concentration, via adjusting for the sulfide concentration.

As a broad-spectrum antimicrobial agent, silver nanoparticles (AgNPs) have been widely used in consumer and medical products. This increased usage of AgNPs translates into increased potential for their release to the environment^1^. This situation has attracted considerable interest in toxicity testing^2^,^3^, and several studies have exposed AgNPs to various organisms, including bacteria^4^,^5^, algae^6^, fungi^7^, *Caenorhabditis elegans*^8^, zebrafish^9^, and human cells^10^,^11^, to verify their specific manner of toxicity and mechanism. Researchers have demonstrated that AgNPs are preferable candidates for antibiotic drugs; however, their physical and chemical characteristics can be impacted by water chemistry properties such as the presence of some anion ions (S^2−^, Cl^−^, SO_4_^2−^)^2^,^12^.

There are two confirmed patterns of action considered as the main toxicity manner of AgNPs: the release of silver ions from the crystalline core of AgNPs, and the promotion of free radical production^13^,^14^,^15^,^16^. Although extensive membrane damage has been observed more severely for AgNPs than for ionic Ag^+^^17^,^18^, the vast majority of toxicity studies of AgNPs have been conducted with respect to the Ag^+^ release mechanism. Some other properties such as the AgNP size, surface coating, surface charge, shape, and solubility have also been considered^2^,^12^,^19^. However, the influence of these factors on the toxicity of AgNPs via indirectly affecting silver ion release remains an open question.

Despite the great amount attention paid to AgNPs based on toxicity applications^20^,^21^,^22^,^23^,^24^,^25^, Xiu *et al.* discovered a new interesting phenomenon that a relatively low concentration (sublethal dose or 12−31% of the minimum lethal concentration for effective Ag^+^) of AgNPs could stimulate *Escherichia coli* activity rather than inhibit it^5^. The same result was proposed by our group, in which *Phanerochaete chrysosporium* (*P. chrysosporium*) was activated by a low concentration (the range is about 0.1–5.0 mg/L) of AgNPs, which promoted its removal capacity to cadmium ions (Table 1)^26^. This result is a great threat to potential applications of AgNPs to exploit the predominant antibacterial action of its products. These findings suggest that in some cases, the antifungal products may stimulate microbial growth but not inhibition as expected. Hence, it is necessary to verify the specific stimulatory effect of AgNPs under both laboratory and real conditions.

Sulfides are widely present in the environment and biological systems, and display strong chemical activity to other matter^27^,^28^,^29^,^30^,^31^. Previous studies have indicated that the sulfidation of Ag surfaces is likely to occur when in contact with various S-bearing molecules^2^. Sulfidation can impact the thermal and electrical conductivity of silver, and even result in the formation of a new shell (such as Ag_2_S) on the surface, which strongly affects the toxicity of AgNPs^32^,^33^. Bone *et al.* demonstrated that more Ag was present as Ag_2_S in the absence of plants^34^. Liu *et al.* demonstrated that sulfidation of AgNPs occurs via two different mechanisms depending on the sulfide concentration^35^. At high sulfide concentrations, sulfidation occurs by direct conversion of AgNPs to Ag_2_S-NPs through a solid−fluid reaction, whereas at lower sulfide concentrations, oxidative dissolution and precipitations seems to be prevalent. Hence, it is necessary to evaluate the effect of sulfides on the toxicity performance of AgNPs in consideration of its antifungal application.

*P. chrysosporium*, as the representative species of white-rot fungi, has been extensively used for its ability to degrade a wide range of organic substrates and to absorb heavy metals in wastewater treatment field^36^,^37^,^38^. The widely use of AgNPs inevitably results in its access to biological water treatment system. Hence, exploitation of the toxic effect of AgNPs on *P. chrysosporium* is an important component in the process of wastewater treatment. In this article, we demonstrated that environmental sulfide induced “toxicity to activation” transformation of citrate-coated AgNPs (Citrate-AgNPs). In the absence of sulfide, a maximum activation concentration (MAC) of Citrate-AgNPs at 3 mg/L was observed. Sulfide addition promoted the initial toxicity of Citrate-AgNPs transformation to activation. Increase of sulfide concentration resulted in higher MAC transfer. Based on this result, we propose a method for controlling the antibacterial activity of Citrate-AgNPs, even at low concentration (3 mg/L), via adjusting for sulfide concentration effectively.

## Results and Discussion

### Synthesis and Characterization of Citrate-AgNPs

Citrate-AgNPs were synthesized according to the method described by Liu *et al.* with slight modifications^39^,^40^. Figure S1 shows that Citrate-AgNPs are spherical in shape with a mean size of 15.09 ± 2.21. The Citrate-AgNPs were found to be nonaggregating in both deionized water and the minimal medium used for assays. The Z-potential was demonstrated to be −47.4 ± 0.7 mV and a typical plasma resonance absorption peak at 396 nm of AgNPs was observed (Fig. S2).

### MAC Transfer and “Toxicity to Activation” Transformation can be Triggered by Sulfide

**Figure 1:**
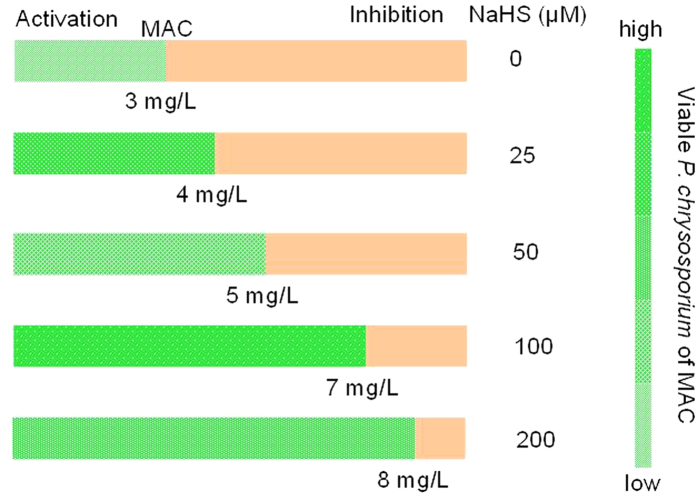
MAC transfer and viable *P. chrysosporium* of MAC induced by NaHS as the sulfide source.

To the best of our knowledge, organic and inorganic matters exhibit different biocompatibilities to cells, which may translate into different effects on the toxicity performance of AgNPs^41^. In this study, the inorganic sulfide source NaHS and the organic sulfide source thioacetamide (TAA) were used as the sulfide sources. To clarify the effect of sulfides on the stimulatory action of Citrate-AgNPs to microbes, we defined the maximum activation concentration of the tested Citrate-AgNPs as the MAC. The MAC of Citrate-AgNPs to *P. chrysosporium* was found to be 3 mg/L without the addition of sulfide (Fig. 1). This result supports previous findings of our group, in which the MAC of Citrate-AgNPs to *P. chrysosporium* was determined to be in the range of 1−5 mg/L^26^. As shown in Fig. 1, the addition of aqueous NaHS promoted substantial MAC changes. The MAC transferred to a higher concentration with the increase of NaHS concentration from 0 to 200 μM as the sulfide source. The transfer degrees induced by various concentration of sulfides (TAA and NaHS) (0, 25, 50, 100, and 200 μM) were 0, 1, 2, 4, and 5 mg/L, respectively. Interestingly, we found that the maximum viability of *P. chrysosporium* at the MAC appeared at 100 μM NaHS but not at 200 μM NaHS; thus, the maximum viability of *P. chrysosporium* at the MAC was not proportional to the NaHS concentration. This different relationship between MAC transfer and maximum viability of *P. chrysosporium* at MAC indicates that the change in MAC transfer was not directly caused by an effect of NaHS on promoting *P. chrysosporium* survival.

**Figure 2:**
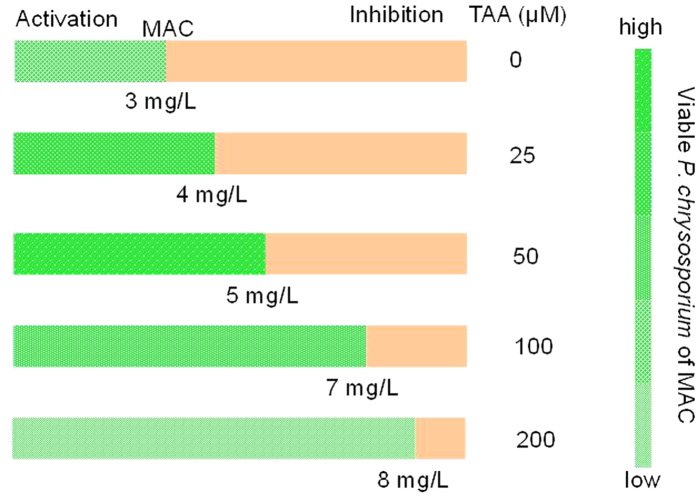
MAC transfer and viable *P. chrysosporium* of MAC induced by TAA as the sulfide source.

Figure 2 shows that TAA produced similar effects to NaHS in terms of the MAC transfer. However, the maximum viability of *P. chrysosporium* at MAC was different, with the best survival observed at a concentration of 50 μM TAA. This result further demonstrates the irrelevance of MAC transfer to changes in *P. chrysosporium* survival promoted by sulfides. In addition, NaHS and TAA showed similar effects on the toxicity of the Citrate-AgNPs. Based on these results, the maximum MAC of 100 μM NaHS, 7 mg/L Citrate-AgNPs and 50 μM TAA, 5 mg/L Citrate-AgNPs were used in subsequent assays.

**Figure 3:**
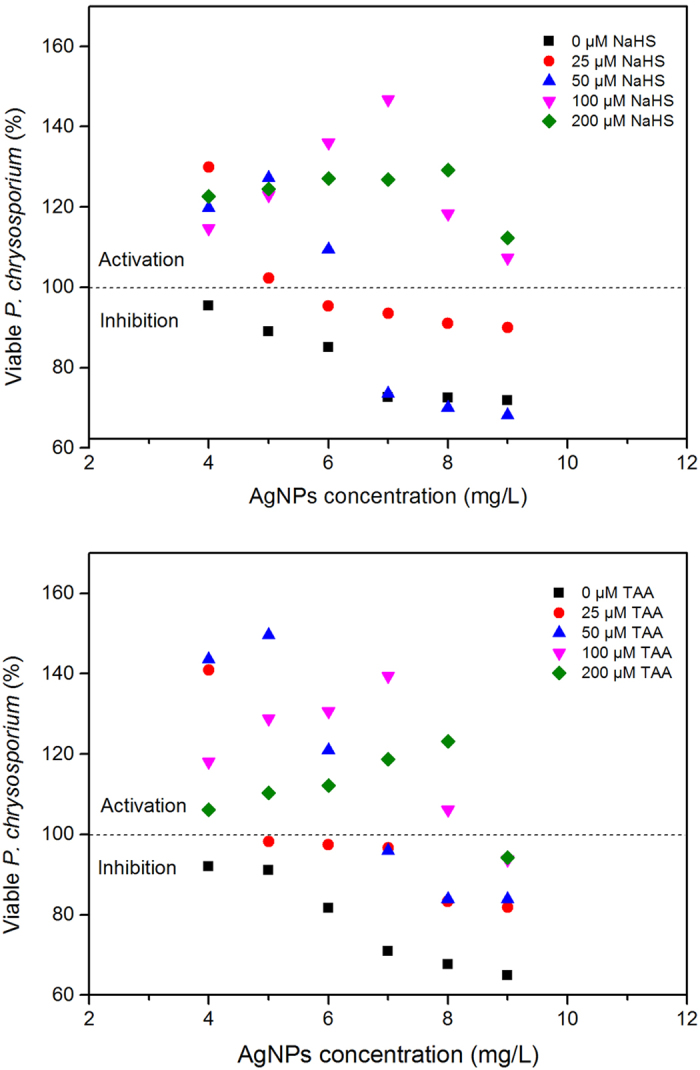
Sulfide induced initial toxic Citrate-AgNPs at higher concentration transfer to activation.

The results described above demonstrated that the sulfides source (both inorganic and organic) induced the activation point transfer of Citrate-AgNPs to high concentration (from 3 mg/L to 8 mg/L with the sulfide concentration added to 200 μM). However, high concentration (>4 mg/L for *P. chrysosporium*) consistently showed excellent inhibition to cell viability in the absence of sulfide. As shown in Fig. 3, this trend resulted in initial toxic Citrate-AgNPs at higher concentration to transfer to an activation effect with the increase of sulfide. This interesting “toxicity to activation” transformation phenomenon prompted us to further evaluate the effect of AgNPs on bacteriostasis.

### Explanation of the Transfer Phenomenon

**Figure 4:**
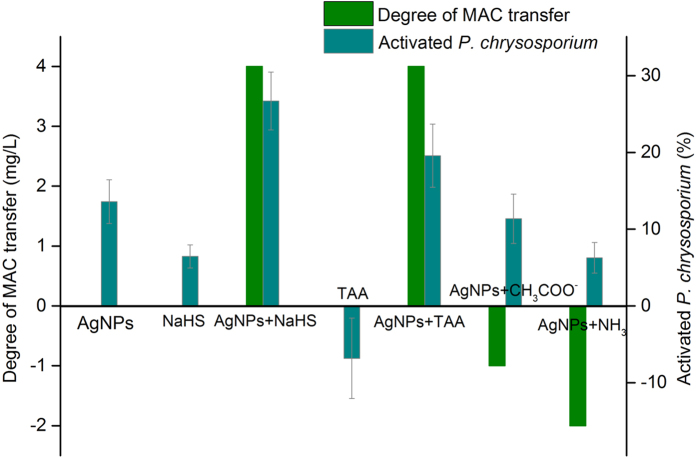
Degree of transfer and P. chrysosporium activation at MAC induced by relevant factors. The concentration was 7 mg/L for AgNPs in the *P. chrysosporium* viability tests, and 100 μM for NaHS, TAA, CH_3_COO^−^ and NH_3_ for all the needed tests.

To discern the contribution of sulfide to the toxicity of Citrate-AgNPs, NaHS, TAA, and equivalent hydrolysis products of TAA, including CH_3_COO^−^ and NH_3_, were tested with and without Citrate-AgNPs in terms of their effects on the transfer degree of MAC and *P. chrysosporium* activation ratio (Fig. 4). The results showed that CH_3_COO^−^ and NH_3_ had no effect on the MAC transfer to higher concentration (CH_3_COO^−^ and NH_3_ cause MAC transfer to lower concentration at 1 mg/L and 2 mg/L, respectively), indicating the essential role of sulfide in promoting the transfer of Citrate-AgNPs. With respect to activation, Citrate-AgNPs and NaHS simultaneously exhibited stimulation effects to *P. chrysosporium*, and CH_3_COO^−^ and NH_3_ had some effect on the toxicity of Citrate-AgNPs. These results suggest that sulfide itself has no effect on the activation of *P. chrysosporium* by Citrate-AgNPs. This may be attributed to the indirect action of sulfide on the toxicity of Citrate-AgNPs. Because the release of Ag^+^ from AgNPs is thought to be one of the main mechanisms governing the toxicity of AgNPs, the MAC would be expected to transfer to a high concentration in the presence of sulfide because of the formation of relatively insoluble Ag_2_S. In the solution, AgNPs easily release Ag^+^ in an aerobic environment (equilibrium constant *K* = 1 × 10^16^, calculated from the ∆G^0^ value of −91.3 kJ/mol at 298 K):^39^


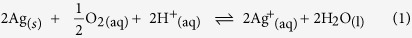


Sulfides react with Ag^+^ of AgNPs surface to form of Ag_2_S as demonstrated by Levard *et al.*^42^, Normally, Ag_2_S dissolves Ag^+^ via the following process. The solubility product constant *K*_sp-Ag2S_ is 5.92 × 10^−51^ for the dissolution process^43^, indicating the lower solubility and toxicity of Ag_2_S^42^,^44^,^45^,^46^,^47^.


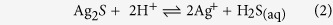


The lower solubility product constant of Ag_2_S indicates that sulfidation could effectively lower the amount of Ag^+^ released and thus reduce the toxicity of Ag_2_S compared to that of AgNPs, accounting for the MAC transfer.

**Figure 5:**
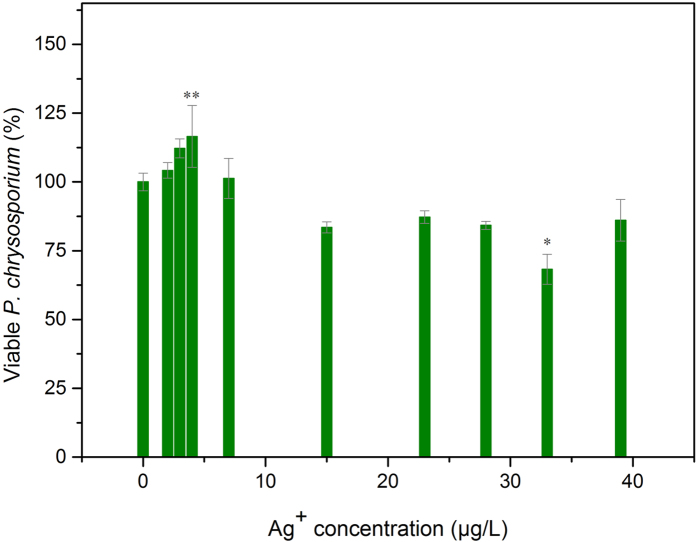
Survival of resting P. chrysosporium cells in buffer solution after 12 h exposure to AgNO3. One asterisk represents significant decrease in viability (*p* < 0.05) relative to unexposed control. A significant stimulatory effect suggestive of hormesis was observed of all treatments, as indicated by two asterisks.

The survival of resting *P. chrysosporium* cells in 2 mM NaHCO_3_ buffer solution was activated in the presence of 3−7 μg/L Ag + after 12 h exposure (the lowest stimulation was less than 3 μg/L in some cases) (Fig. 5). Ag^+^ concentration ~4 μg/L exhibited a significant stimulatory effect compared to the unexposed control group.

**Figure 6:**
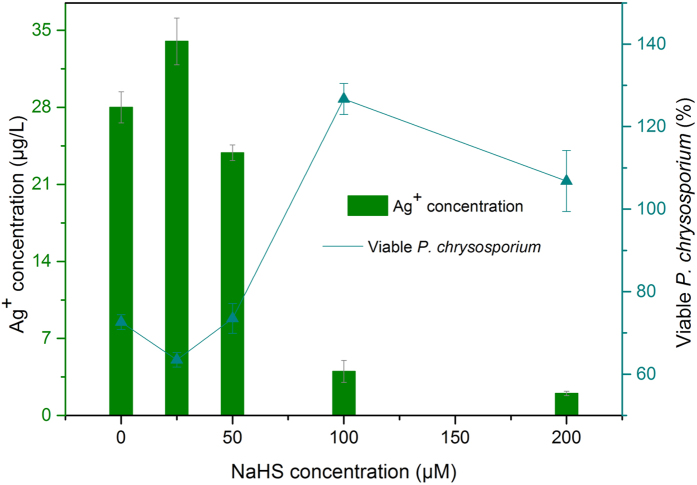
The concentration of residue Ag+ and viable P. chrysosporium at MAC with various NaHS. The concentration of Citrate-AgNPs was 7 mg/L.

**Figure 7:**
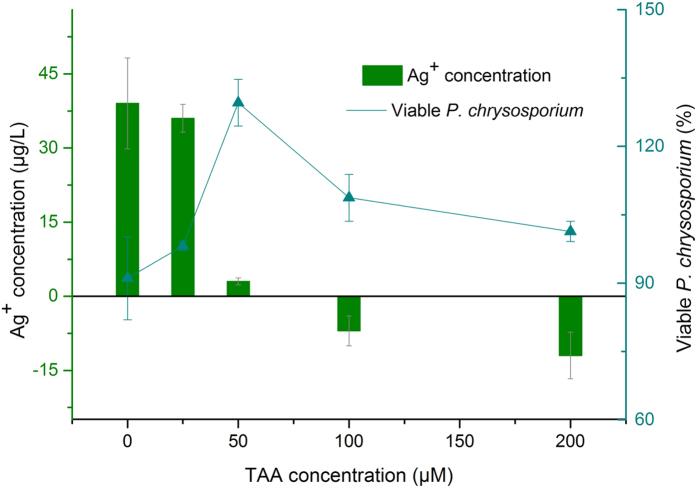
The concentration of residue Ag+ and viable P*. chrysosporium* at MAC with various TAA. The concentration of Citrate-AgNPs was 5 mg/L.

The Ag^+^ residue in the solution was determined to be conformed to the activation concentration (3−7 μg/L for Ag^+^) after exposure to 7 mg/L Citrate-AgNPs, 100 μM NaHS (Fig. 6), which was the condition for the highest viability of *P. chrysosporium* at MAC, as shown in Fig. 1. Simultaneously, the addition of 5 mg/L Citrate-AgNPs and 50 μM TAA showed an optimal stimulation concentration of Ag^+^ (3 μg/L) (Fig. 7). Higher and lower sulfide concentrations would cause the Ag^+^ residue to be outside of the activation range. These Ag^+^ concentrations range well explained the variation in *P. chrysosporium* viability at MAC and the effect of sulfide on Ag^+^ release. This finding is in line with the interpretation that the toxicity of AgNPs is mainly caused by Ag^+^ release. A significant decrease in the surface plasma resonance of Citrate-AgNPs was also observed after the addition of sulfide source (Figs S3 and S4); that may be attributed to stable material production on the surface (Ag_2_S, as proposed above)^40^. As a result, the Ag^+^ release was limited (Figs 6 and 7), the toxicity process of AgNPs based on Ag^+^ was interfered. These results together demonstrate that the sulfide did indeed have an effect on Ag^+^ release.

### Tests of Antibacterial Activity of Citrate-AgNPs and Antifungal/Antibacterial Activity Biologically Synthesized AgNPs

To assess the effect of sulfide on Citrate-AgNPs toxicity on bacteria, gram-negative bacterium *Escherichia coli* (*E. coli*) was employed for the toxicity assays. As shown in Fig. S5, sulfide induced the analogous trend in antibacterial activity. These results indicate that the methods could be used in antibacterial application simultaneously.

In addition, we tested the effect of sulfide on biologically synthesized AgNPs toxicity on *P. chrysosporium* and *E. coli* (Table S1). No obvious MAC transfer was observed on this biologically synthesized AgNPs. The S–H groups of the AgNPs surface forming an Ag–S bond as demonstrated by Sanghi *et al.*^48^, has a similar effect of TAA and NaHS, which may cause the unobvious MAC transfer.

### Potential Application of MAC Transfer in Antibacterial Use

A low dose of AgNPs has a hormesis effect on microbial and animal cell viability (Table S2). This effect has been interpreted as overcompensation for the exposure, because low doses of toxicants can activate the repair mechanisms of cells against the toxicant^5^,^49^. As shown in Table S2, the hormesis effect normally occurred in the low concentration range of AgNPs, which was regarded as an effective district for the hormesis effect. Lower and higher concentrations resulted in inhibition of cell viability.

**Figure 8:**
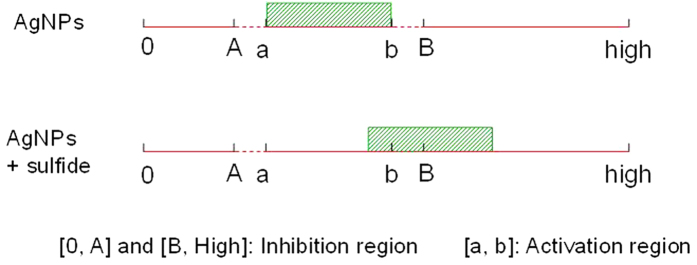
chematic for hormesis effect theory of AgNPs. The region of [0, high] indicate the concentration of AgNPs from 0 to higher. The slash box transfer to higher concentration, which indicates the activation region transferring to higher concentration after addition of sulfide (cysteine or Na_2_S).

To illustrate the hormesis effect more clearly, we constructed the graph presented in Fig. 8. Under certain conditions, the concentration region [a, b] was considered to be the effective district of the hormesis effect, whereas [0, A] and [B, high] were regarded as the inhibition districts. According to the literature, [A, a] and [b, B] likely represent uncertain districts for unreported activation or even inhibition effects^50^,^51^. Figure 8 indicates that the region [a, b] is disadvantageous to the antibacterial effect of AgNPs. External action would be needed to maintain the concentration within the region of [0, A] or [B, high] to ensure the effective antibacterial activity of AgNPs.

As described above, sulfide can induce the MAC transfer to a higher concentration, shifting the initial low activation or even inhibition concentration ([B, high]) toward high stimulation (Fig. S6). In other words, the main toxic and activation effects of AgNPs are attributed to the released Ag^+^ concentration. Hence, the addition of sulfide could lead to a change to inhibition when in its activated state ([a, b]) due to the certain range of the activation district. The usual concentration of AgNPs used in antibacterial agents is low ( < 5.0 mg/L)^52^, so that the antibacterial activity is located at the stimulation district. Therefore, sulfide addition could effectively control the antibacterial ability of AgNPs. For example, when point A was 2.5 mg/L and point B was 3.5 mg/L for AgNPs concentration, the initial MAC was 3 mg/L AgNPs. The simple addition of 25 μM TAA would cause a MAC transfer to 4 mg/L (Fig. 3). Under the precondition of a uniform sulfidation rate, point A and B would change to 3.5 mg/L and 4.5 mg/L, respectively. The initial MAC of 3 mg/L is therefore transferred to inhibition from the activation state. With continuous addition of sulfide, point A would further transfer to a higher concentration, retaining a larger inhibition range of [0, A]. This proposed strategy could be effective for bacteriostasis applications to enhance the sterilizing effect of AgNPs at a concentration of antimicrobial reagents within stimulation region.

Although this study was conducted with the addition of sulfide, our results may provide an effective approach for effectively controlling and utilizing the hormesis effect of AgNPs in antibacterial applications. Some substances may also interfere with AgNPs bacteriostasis. To avoid activation in the sterilization process, a new convenient method may be developed based on this result.

## Conclusions

In summary, this study proposed the MAC transfer of Citrate-AgNPs under the induction of inorganic sulfide source (NaHS) or organic sulfide source (thioacetamide, TAA). Sulfide (TAA or NaHS) addition promoted the MAC transfer to higher AgNPs concentration, which results in the initial inhibition concentration of Citrate-AgNPs changing to activation as “toxicity to activation” transformation pattern. Higher sulfide concentration results in larger transfer of the site. The best activation concentration of sulfide was 100 μM for NaHS and 50 μM for TAA, which achieved maximal viability of *P. chrysosporium* at ~126.7% and ~129.6%, respectively. In addition, the application of this BSP transfer effect in improving antibacterial activity was proposed. The antibacterial activity of AgNPs can be controlled, even at low concentration, via adjusting for the MAC transfer effectively.

## Methods

### Synthesis of Citrate-AgNPs

A 59.8 mL solution containing 0.6 mM trisodium citrate and 0.4 mM NaBH_4_ was prepared in double distilled water and stirred vigorously in an ice bath. Upon addition of 0.48 mL, 23.5 mM AgNO_3_ under stirring, the colour of solution turned yellow, indicating the formation of Citrate-AgNPs. After 3 h of additional stirring at room temperature, the soluble byproducts were removed by centrifugal ultrafiltration (molecular weight cutoff of 1000), and the Citrate-AgNPs were washed with double distilled water. The morphology and EDAX spectrum are provided in Fig. S7.

### *Phanerochaete chrysosporium* identification and cultivation

To confirm *P. chrysosporium* isolation, the genomic DNA of *P. chrysosporium* was extracted using the Ezup Column Fungi Genomic DNA Purification Kit following the manufacturer’s instructions (Sangon Biotech Co., Ltd., Shanghai, China). The 18S rDNA gene sequence of the genomic DNA was amplified by PCR using primer sets of NS1 (5′-GTAGTCATATGCTTGTCTC-3′) and NS6 (5′-GCATCACAGACCTGTTATTGCCTC-3′). PCR amplification was performed in a 25 μL reaction mixture containing 0.5 μL of each primer (10 μM), 0.5 μL of template DNA (20–50 ng/μL), 0.2 μl of Ex Taq (5 Uμl^−1^), 2.5 μL of 10 × PCR buffer with mg^2+^, 1.0 μL of dNTP (2.5 mM each), and sterile distilled water to a final volume of 25 μL. PCR cycling was performed under the following conditions: an initial denaturing step at 94 °C for 4 min, followed by 30 cycles of denaturation at 94 °C for 45 s, annealing at 55 °C for 45 s, and elongation at 72 °C for 1 min. Finally, an extension step was performed at 72 °C for 10 min. PCR products were verified by agarose gel electrophoresis (1.0% weight/volume agarose) with ethidium bromide staining and visualized using an ultraviolet (UV) transilluminator. After that, the target gene fragments were purified using SanPrep Column DNA Gel Extraction Kit (Sangon Biotech Co., Ltd.) in accordance with the manufacturer’s instructions. And then, the purified products were sequenced by the Sangon Biotech Co., Ltd. The 18S rDNA gene sequences obtained were analyzed with the BLAST program of the GenBank database at the National Center for Biotechnology Information (NCBI) website (http://www.ncbi.nlm.nih.gov/). The results show that the 18S rDNA sequences of *P. chrysosporium* shared 100% and 99% similarity with *Phanerochaete* sp. Y6 (accession No. DQ438911) and *P. chrysosporium* KSR2 (accession No. KJ606692) respectively.

*P. chrysosporium* strain BKMF-1767 (CCTCC AF96007) used in this study was obtained from the China Center for Type Culture Collection (Wuhan, China). Stock cultures were maintained on malt extract agar slants at 4 °C. Aqueous suspensions of fungal spores were inoculated into Kirk’s liquid culture medium and incubated at 37 °C in an incubator according to previous report^53^. After 2 days of growth in liquid culture, the mycelia were harvested by centrifugation at 10000 × g for 5 min, washed three times with 2 mM sodium bicarbonate buffer solution, and resuspended in the same buffer to make the *P. chrysosporium* stock solution. Sodium bicarbonate can buffer the system at relatively low ionic strength (which promotes nanoparticle coagulation) compared to other bacteria media, and was chosen as the exposure medium to avoid ligands that could bind with Ag^+^/AgNPs and promote precipitation or other confounding effects (AgNPs/Ag^+^) toxicity assays in this work were below Ag_2_CO_3_ precipitation potential (*K*sp = 0.81 × 10^−12^)^5^,^13^.

### Dose-response assay of Citrate-AgNPs on *P. chrysosporium*

Equivalent mycelia (0.4 g) were added respectively into test tubes to achieve an identical cell concentration. Before toxicity tests, Citrate-AgNPs stock solution was diluted in 2 mM sodium bicarbonate buffer to obtain different concentrations. Aqueous Citrate-AgNPs and sulfide were mixed and equilibrate with *P. chrysosporium* in order to begin the toxicity test. After 12 h, mycelia were centrifugation to remove residual AgNPs and Ag^+^.

Cell viability assays were operated according to Luo *et al.* and Chen *et al.*^54^,^55^, 0.2 g *P. chrysosporium* pellets were mixed with 1 mL MTT solution (5 g/l) and incubated at 50 °C for 1 h. The reaction was stopped by adding 0.5 mL hydrochloric acid (1 M) to the mixture. The mixture was centrifuged (10,000 × g, 5 min), the supernatant was discarded, and the pellets were agitated in 6 ml propan-2-ol for 2 h at 25 °C. The centrifugation process was repeated and the absorbance of the supernatant was recorded at 534 nm using spectrophotometer (Model UV-2550, Shimadzu, Japan).

For Ag^+^ testes, the solution was ultrafiltration centrifuged at 10000 × g. The Ag^+^ concentration of filtrate was then determined using ICP-OES (IRIS Intrepid II XSP, Thermo Electron Corporation, USA)

### Statistical analyses

Whether differences between treatments were statistically significant was determined using Student’s *t* test at the 95% confidence level (Statistical Program for Social Sciences 19.0). All measurements are reported as mean ± one standard deviation with three replicates.

### Associated Content

SUPPORTING INFORMATION AVAILABLE: Methods of dose-response assay of Citrate-AgNPs on *E. coli*, biosynthesis of AgNPs, and dose-response assay of biologically synthesized AgNPs on *P. chrysosporium* and *E. coli*. TEM, UV-vis characterization of Citrate-AgNPs (Figs S1, S2). Sulfide induced decrease in the surface plasma resonance of Citrate-AgNPs (Figs S3, S4). Sulfide effect of Citrate-AgNPs toxicity on *E. coli* (Fig. S5) and biologically synthesized AgNPs on *P. chrysosporium* and *E. coli* (Fig. S6). Graph for sulfide effect on AgNPs toxicity of initial inhibition state (Fig. S6). HAADF-STEM image of Citrate-AgNPs and EDAX spectrum (Fig. S7). Development of AgNPs hormesis effect (Table S2).

## Additional Information

**How to cite this article**: Guo, Z. *et al.* Activity Variation of *Phanerochaete chrysosporium* under Nanosilver Exposure by Controlling of Different Sulfide Sources. *Sci. Rep.* **6**, 20813; doi: 10.1038/srep20813 (2016).

## Supplementary Material

Supplementary Information

## Figures and Tables

**Table 1:** Proposed AgNPs hormesis effect to microbe.

target organisms	reported maximum activation point (mg/L)	optimal activation range^a^ (mg/L)	AgNPs category	reference
*E. coli*	2.2	1.0–4.0	PEG-AgNPs-3 nm	5
	1.8	0–3.5	PEG-AgNPs-5 nm	
	2	0–4.5	PEG-AgNPs-11 nm	
*E. coli*	16.4	5.0–35.0	PVP-AgNPs-20 nm	5
	5.7	0–10.0	PVP-AgNPs-40 nm	
	6.7	10.0–30.0	PVP-AgNPs-80 nm	
P. chrysosporium	1	0.1–5.0	Citrate-AgNPs-24 nm	26

^a^estimated from the figure of the literature.
