# Supplementary material for: Activity Variation of Phanerochaete chrysosporium under Nanosilver Exposure by Controlling of Different Sulfide Sources
Source: Sci Rep. 2016 Feb 11;6:20813. doi: 10.1038/srep20813 (PMC4749979; doi:10.1038/srep20813)
Supplement: Supplementary Information [file srep20813-s1.pdf]

Activity Variation of *Phanerochaete chrysosporium* under Nanosilver Exposure by Controlling of Different Sulfide Sources

Zhi Guo,[†,‡] Guiqiu Chen,[*,†,‡] Lingzhi Liu,[†,‡] Guangming Zeng,[*,†,‡] Zhenzhen Huang[†,‡] Anwei Chen,[§] Liang Hu[†,‡]

[†]College of Environmental Science and Engineering, Hunan University, Changsha 410082, P.R. China

[‡]Key Laboratory of Environmental Biology and Pollution Control (Hunan University), Ministry of Education, Changsha 410082, P.R. China

[§]College of Resources and Environment, Hunan Agricultural University, Changsha 410128, P.R. China

[*]Corresponding Author. Phone: +86 731 88822829. Fax: +86 731 88823701. E-mail: gqchen@hnu.edu.cn (G.C.); zgming@hnu.edu.cn (G.Z.).

Dose-response assay of Citrate-AgNPs on *Escherichia coli*. The toxicity tests of AgNPs on *Escherichia coli* (*E. coli*) were operated according to the previous report.[1,2] Briefly, the harvested *E. coli* was diluted to a concentration of OD_{600} = 0.1 with 2 mM sodium bicarbonate buffer. AgNPs stock solution was diluted with the same buffer to obtain different concentrations. Then, AgNPs solution was added with *E. coli* and sulfide, and incubating for 6 h. After the incubation, the *E. coli* was centrifuged to remove the supernatant in tube. A volume of 0.5 mL of the thawed stock MTT solution was added to each sample at different treated, and samples were incubated at 37 ℃ for 1 h. Acid-isopropanol (3 mL of 0.04 M HCl in isopropanol) was added to all of the samples and mixed thoroughly to dissolve the dark blue crystals. After incubation for 2 h at room temperature to ensure that all of the crystals were dissolved, the absorbance was measured at a wavelength of 570 nm using a UV-vis spectrophotometer. All of the samples were analyzed in triplicate.

Biosynthesis of AgNPs and dose-response assay. AgNPs were synthesized by fungal proteins of *Coriolus versicolor* according to Sanghi et al.[3] Briefly, around 12 g of biomass (fresh weight) was brought in contact with 5 mM $AgNO_3$ final concentration in a 150 ml Erlenmeyer flash and agitated at 37 ℃ in dark. The color of the media solution turned light brown in 48 h indicating the formation of AgNPs.

The dose-response assay of biologically synthesized AgNPs on *P. chrysosporium* and *E. coli* were operated similar to the toxicity tests of Citrate-AgNPs.

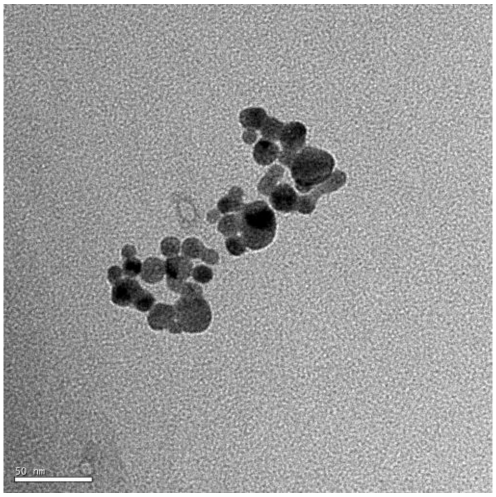

Figure S1. TEM of Citrate-AgNPs.

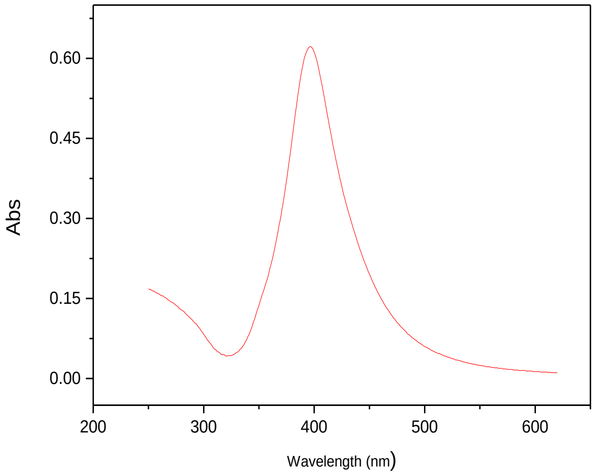

Figure S2. Plasma resonance absorption of Citrate-AgNPs.

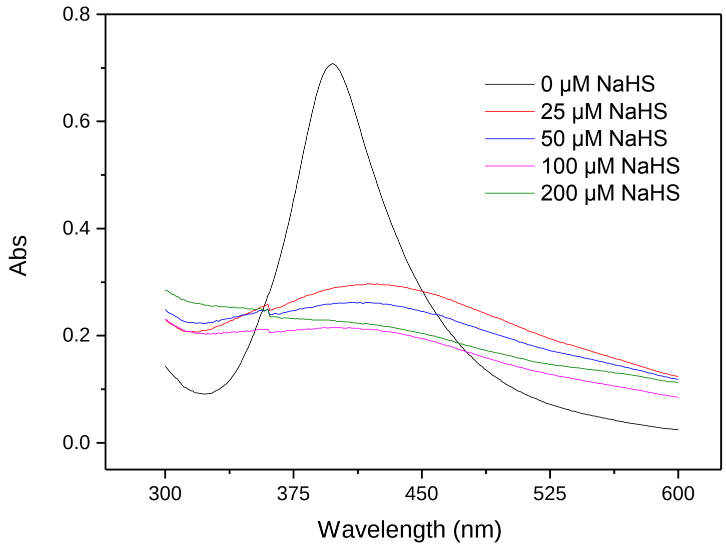

Figure S3. Surface plasma resonance decrease of AgNPs induced by NaHS.

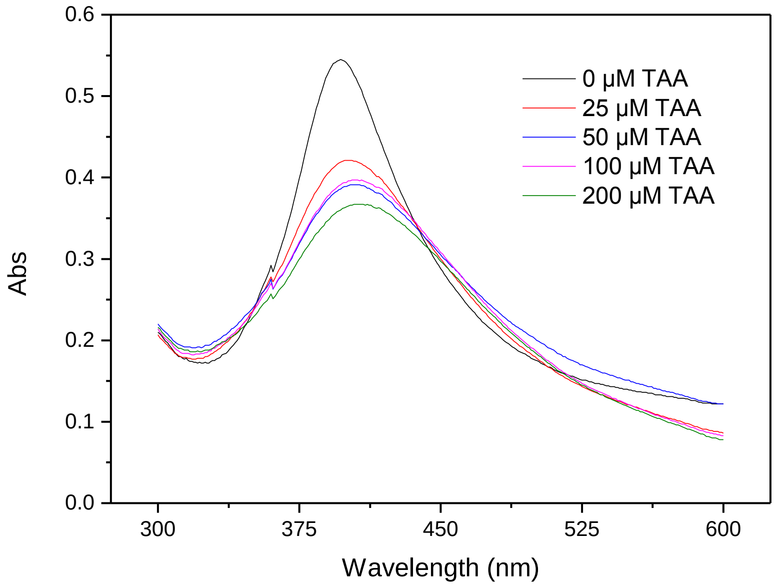

Figure S4. Surface plasma resonance decrease of AgNPs induced by TAA.

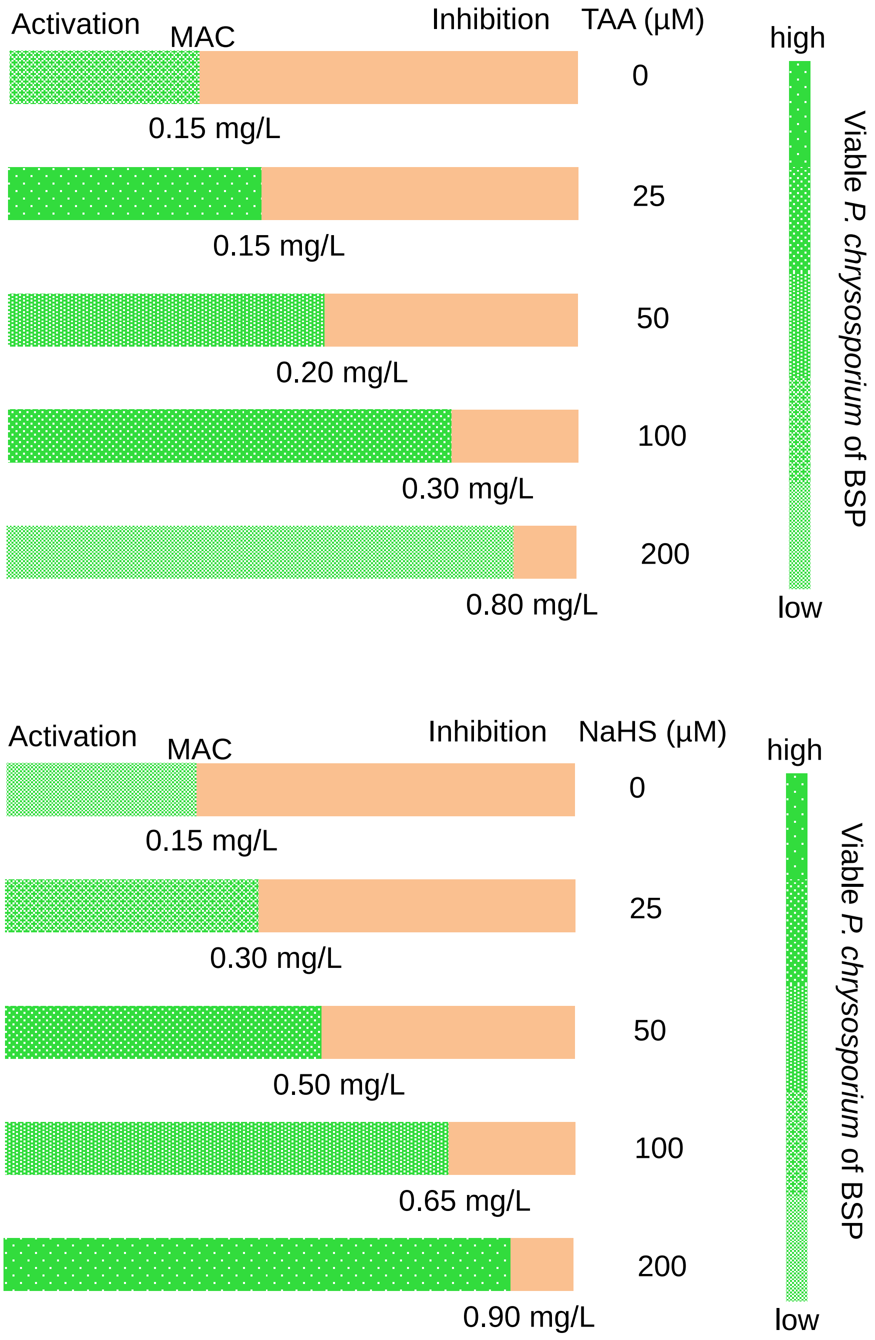

Figure S5. MAC transfer and viable *E. coli* of MAC induced by TAA and NaHS as the sulfide source.

Table S1. MAC transfer of *P. chrysosporium* and *E. coli* induced by TAA and NaHS as the sulfide source.

TAA (μM)	0	25	50	100	200
MAC (mg/L)	9.5	9.5	9.5	10	10
NaHS (μM)	0	25	50	100	200
MAC (mg/L)	9.5	10	10	10	10

Table S2. Development of AgNPs hormesis effect.

target organisms	Range of concentrations	Hormetic responss	reference
Spermatogonial stem cell line C18-4	5 – 100 mg/L	Low dose activation and high dose inhibition	4
Peripheral blood mononuclear cells PBMCs	1 – 30 ppm	Low dose activation and high dose inhibition	5
Human skin carcinoma cell line A431 and human fibrosarcoma cell line HT-1080	0.39 – 25 mg/L	For A431 cells, activation at 1.56 – 6.25 mg/L and inhibition at concentrations ≤ 0.78 and ≥ 12.5 mg/L. For HT-1080 cells, activation at 0.78 – 6.25 mg/L and inhibition at concentrations ≤ 0.39 and ≥ 12.5 mg/L	6
Human hepatoma derived cell line HepG2	0.1 – 3 mg/L	Low dose activation and high dose inhibition	7
E. coli	0 – 9 mg/L (PEG-AgNPs)	Activation at 12−31% of the MLC for Ag^+	8
E. coli	0 – 70 mg/L (PVP-AgNPs)	Activation at 12−31% of the MLC for Ag^+	8

MLC: minimum lethal concentration.

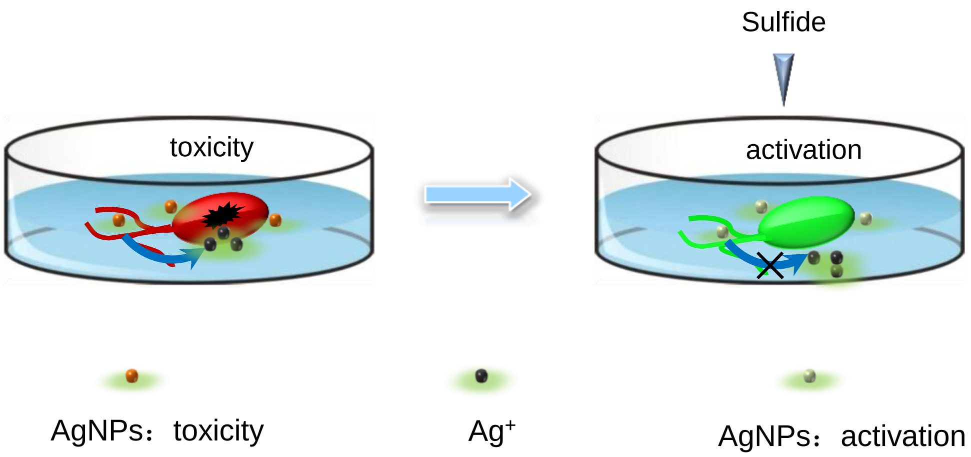

Figure S6. Graph for sulfide effect on AgNPs toxicity of initial inhibition state.

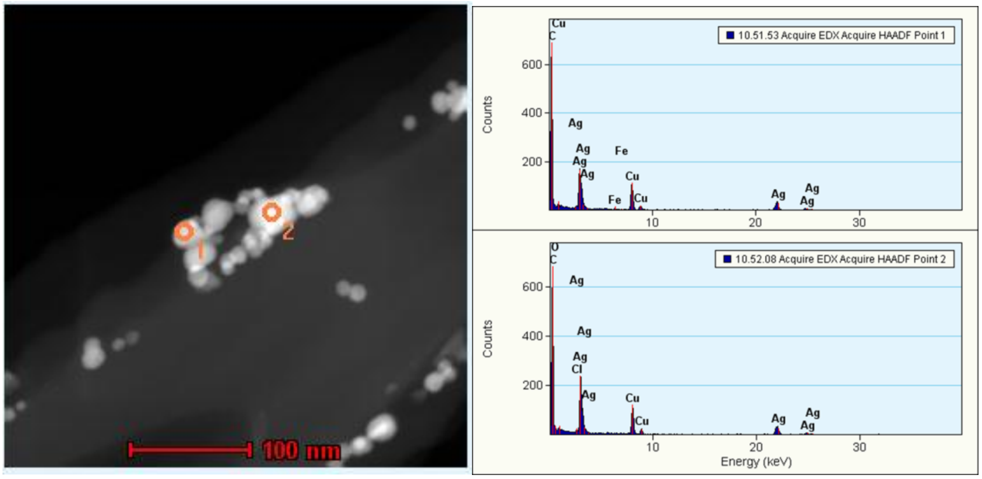

Figure S7. HAADF-STEM image of Citrate-AgNPs and EDAX spectrum from the point 1 and 2.

REFERENCES

1. Wang, H., Cheng, H., Wang, F., Wei, D. & Wang, X. An Improved 3-(4,5-dimethylthiazol-2-yl)-2,5-diphenyl Tetrazolium Bromide (MTT) Reduction Assay for Evaluating the Viability of *Escherichia coli* Cells. *J. Microbiol. Methodss* **8**, 330–333 (2010).

2. Liu, Y. & Nair, M. G. An Efficient and Economical MTT Assay for Determining the Antioxidant Activity of Plant Natural Product Extracts and Pure Compounds. *J. Nat. Prod.* **73**, 1193–1195 (2010).

3. Sanghi, R. & Verma, P. Biomimetic Synthesis and Characterisation of Protein Capped Silver Nanoparticles. *Bioresour. Technol.* **100**, 501–504 (2009).

4. Braydich-Stolle, L., Hussain, S., Schlager, J. J. & Hofmann, M. C. In Vitro Cytotoxicity of Nanoparticles in Mammalian Germline Stem Cells. *Toxicol. Sci.* **88**, 412−419 (2005).

5. Shin, S. H., Ye, M. K., Kim, H. S. & Kang, H. S. The Effects of Nano-Silver on the Proliferation and Cytokine Expression by Peripheral Blood Mononuclear Cells. *Int. Immunopharmacol* **7**, 1813−1818 (2007).

6. Arora, S., Jain, J., Rajwade, J. M. & Paknikar, K. M. Cellular Responses Induced by Silver Nanoparticles: In Vitro Studies. *Toxicol. Lett.* **179**, 93−100 (2008).

7. Kawata, K., Osawa, M. & Okabe, S. In Vitro Toxicity of Silver Nanoparticles at Noncytotoxic Doses to Hepg2 Human Hepatoma Cells. *Environ. Sci. Technol.* **43**, 6046−6051 (2009).

8. Xiu, Z. M., Zhang, Q. B., Puppala, H. L., Colvin, V. L. & Alvarez, P. J. J. Negligible Particle-Specific Antibacterial Activity of Silver Nanoparticles. *Nano Lett*. 12, 4271−4275 (2012).
